# Evaluation of an online Diabetes Needs Assessment Tool (DNAT) for health professionals: a randomised controlled trial

**DOI:** 10.1186/1745-6215-10-63

**Published:** 2009-07-30

**Authors:** Sara Schroter, Dean Jenkins, Rebecca Playle, Kieran Walsh, Courtenay Probert, Thomas Kellner, Gerhard Arnhofer, David Owens

**Affiliations:** 1 BMJ Editorial Office, BMJ Group, BMA House, Tavistock Square, London WC1H 9JR, UK; 2 BMJ OnExamination, Cardiff Medicentre, Heath Park, Cardiff, CF14 4UJ, UK; 3 South East Wales Trials Unit, Department of Primary Care & Public Health, Cardiff University, 7th Floor, Neuadd Meirionnydd, Heath Park, Cardiff, CF14 4YS, UK; 4 BMJ Learning, BMJ Publishing Group, BMA House, Tavistock Square, London WC1H 9JR, UK; 5 BMJ OnExamination, Cardiff Medicentre, Heath Park, Cardiff, CF14 4UJ, UK; 6 MSD RBSC GmbH, Richard Reitzner Allee 1, 85540 Haar, Germany; 7 Merck Sharp & Dohme Limited, Hertford Road, Hoddesdon, EN11 9BU, UK; 8 Diabetes Research Unit, 1st Floor Academic Centre, University Hospital Llandough, Penlan Road, Penarth, CF64 2XX, UK

## Abstract

**Background:**

Continuous medical education is traditionally reliant to a large extent on self-directed learning based on individuals' perceived learning priorities. Evidence suggests that this ability to self-assess is limited, and more so in the least competent. Therefore, it may be of benefit to utilise some form of external assessment for this purpose. Many diabetes educational programmes have been introduced, but few have been assessed for their benefit in a systematic manner. As diabetes is an increasingly prevalent disease, methods for the dissemination and understanding of clinical guidelines need to be explored for their effectiveness. This paper describes the study design of a randomised controlled trial to evaluate the effectiveness of using an interactive online Diabetes Needs Assessment Tool (DNAT), that builds a learning curriculum based on identified knowledge gaps, compared with conventional self-directed learning. The study assesses the effect of these interventions on health professionals' knowledge of diabetes management, evaluates the acceptability of this process of learning and self-reported changes in clinical practice as a result of this novel educational process.

**Methods:**

Following a baseline assessment, participants will be randomised to undergo a 4-month learning period where they will either be given access to the diabetes learning modules alone (control group) or a Diabetes Needs Assessment Tool (DNAT) plus the diabetes learning modules (intervention group). On completion of the DNAT, a personalised learning report will be created for each participant identifying needs alongside individualised recommendations of the most appropriate learning modules to meet those requirements. All participants will complete a Diabetes Knowledge Test before and immediately after the allocated learning and the primary outcome will be the state of knowledge at 4 months. Learners will also be surveyed immediately after the learning period to assess the acceptability of the learning formats and the perceived usefulness and usability of the materials. After a further month, all learners will receive a series of questions to evaluate self-reported changes in clinical practice as a result of this educational experience and asked to include specific examples of any changes in their diabetes care practice.

**Trial registration:**

Current Controlled Trials ISRCTN67215088

## Background

Doctors need ongoing learning in order to stay up to date. Therefore, they need a method of continuous professional development that is convenient and practical which enables them to meet their personalised needs. Online learning provides learners the opportunity to personalise their learning, for example, the order in which they read material, the speed of their learning, where and when they choose to learn.[1] In agreement with previous reviews, [1-3] a more recent systematic review and meta-analysis [4] of the effects of internet-based learning involving health professionals in a wide range of learning contexts and clinical areas reaffirmed that internet-based learning can be as effective as more traditional methods of learning. Major positive effects were found compared with no intervention for both knowledge outcomes, skills, learner behaviour and patient effects. This suggests that future research should focus only on direct comparisons between the different types of internet based interventions.

Continuous medical education is traditionally reliant to a large extent on self-directed learning based on the individual's perceived priorities for learning. Evidence suggests that this ability to self-assess is limited, and more so in the least competent.[5] There is also evidence that practitioners prefer to focus on topics which they are already familiar with and avoid less familiar topics.[6] Current thought is that interactive CME activities that help individuals recognise a "teachable moment" and an opportunity for learning can help encourage engagement in the learning and motivate learners to pursue the activity.[7] While there are methods to help you discover your knowledge and skills gaps, there is no one definitive methodology. Using a variety of methods often gives a better overall view.[8] Therefore it may be of benefit to have some form of formal external assessment.[9] Exclusive reliance on a formal needs assessment might discourage creativity and professionalism,[10] but it could be an important part of the process.

Miller [11] has defined the ideal stages of the development of physician skills. The first level is "declarative knowledge" i.e. the acquisition and interpretation of facts. This is followed by "procedural knowledge" where individuals can describe how to do something but may not be able to actually do it. The third level is about demonstrating that you know how to do what you have learned, i.e. developing "competence". The fourth level involves individuals using the competence they have developed in their actual clinical practice ("performance"). Moore et al's[7] conceptual framework of an approach to continuous planning and assessment in continuing medical education builds on Miller's[11] model to incorporate an assessment on improvements in patients' health status and finally the health status of the population. However, Curran and Fleet [12] reviewed the types of outcome measures used in evaluative research in web-based CME and found that most research has focused on participants' satisfaction and not on change in clinical practice or impact on patient and health outcomes. While assessing the effect on patient's health status can be hampered by ethical constraints in gaining access to data,[7] assessing whether learners have tried to use their competence in practice seems crucial in an assessment of the effectiveness of a learning intervention.

As diabetes is an increasingly prevalent disease, methods for the dissemination and understanding of clinical guidelines need to be explored for their effectiveness. In educational research on diabetes much of the focus has been on the mode of delivery of the education with mixed results from traditional method of small group teaching to larger structured learning programmes.[13,14] Internet-based interventions in diabetes have been shown to increase guidelines knowledge[15,16] and compliance.[17] Many education programmes have been examined but few in a systematic manner and across wide geographic areas involving large numbers.

There is no previous randomised controlled trial (RCT) evaluating the effectiveness of different formats of administering online learning materials in the field of diabetes on knowledge and subsequent change in clinical practice. Therefore, we have designed an RCT of two different formats of online learning for health professionals, i.e. an interactive online learning tool with access to relevant learning modules (active intervention group) compared with access to relevant learning modules alone (control group) to assess effectiveness in terms of diabetes knowledge, satisfaction and self-reported change in clinical practice. We describe here the design of this randomised controlled trial.

## Methods

### Design

This is a randomised controlled trial to evaluate the effectiveness of using an online interactive learning tool, Diabetes Needs Assessment Tool (DNAT), in conjunction with a learning management system (LMS) to improve health professionals' knowledge of how to manage diabetes; to evaluate the acceptability of this process of learning; and self-reported changes in clinical practice as a result of this educational process.

### Participants

Volunteers will be recruited through targeted emails and advertisements inviting English and German speaking practicing doctors and nurses to take part in an educational research project. Interested individuals will be asked to complete an eligibility questionnaire online (see inclusion/exclusion criteria) and those deemed eligible will be given the opportunity to register for the trial. We have produced two parallel websites, one for English speaking participants and the other for German speaking participants. Participants are able to choose whether they want to receive the materials in English or German.

Participants will be informed that the time spent on learning activities for the project will contribute to their Continuing Professional Development (CPD) requirements and that they will receive a personalised certificate of learning on completion of the course. Participants will be told that they will receive their test results and correct answers, and access to the most effective learning package at the end of the study free of charge. In addition, a choice of access to one of three BMJ knowledge related products (English speaking participants) and some QUAIME AG online learning modules (German speaking participants) will be provided.

#### Inclusion and exclusion criteria

On registering for the study, potential participants will be asked a series of screening questions ensuring that only eligible participants are recruited i.e. participants must be either English or German speaking practising doctors or nurses managing at least one patient with diabetes per week. Note – German nurses, except diabetes nurses and assistants for family physicians, will be excluded from taking part as they are not licensed to receive training where information about pharmacological treatment is included.

#### Ethical approval and trial registration

The Research Ethics Committee for Wales confirmed that the study does not require full ethical review (personal communication 07 January 2009). The research will be carried out in compliance with the Helsinki Declaration. All participants will be asked to give their consent to take part in registration. The trial was registered with Current Controlled Trials before any participants were recruited (Current Controlled Trials ISRCTN67215088). The study adheres to the Good Publication Practice guidelines for pharmaceutical companies.

### Assessments and procedures

**Figure 1:**
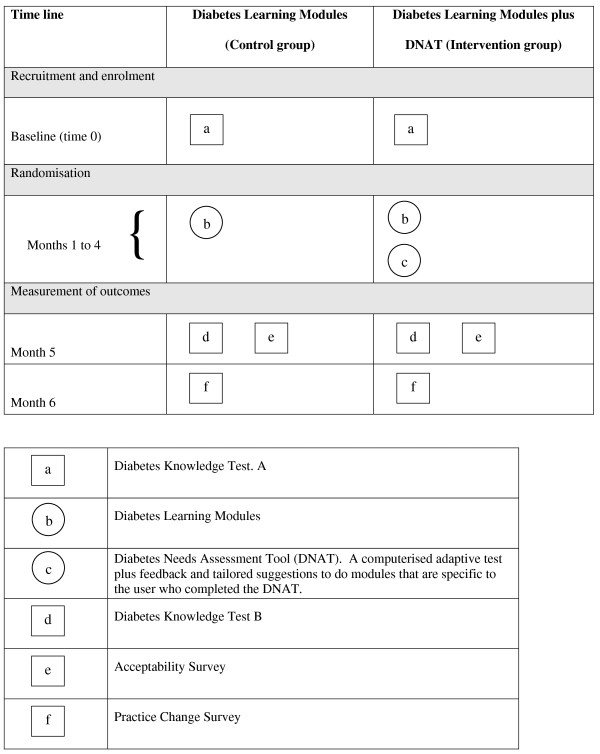
**Schematic representation of components of the intervention and assessment points**.

All assessments, learning materials, and surveys will be administered to participants online at the study website. Participants will be asked to give their consent to take part in a randomised controlled trial designed to test the effectiveness of different learning materials and methods and will be informed that they will be required to use the online learning materials for at least five hours during the 4-month learning period. Figure 1 is a schematic representation of the key components of the trial.

Once registered, participants will be asked to complete the first Diabetes Knowledge Test before being randomised to a study group. At the start of the 4-month learning period they will be directed to their randomly allocated learning materials. Learners in both groups will receive the same six automated reminders to look at the learning materials during the learning period (4, 8, 10, 12, 14, and 16 weeks after the start of the learning period). The intervention group will not be reminded to look specifically at the DNAT during the learning period.

Immediately after the learning period, access to the learning materials will be closed and all learners will be asked to complete the second Diabetes Knowledge Test (Test B) and the Acceptability Survey. After a further month, all learners will be asked to complete the Practice Change Survey. The administration of these tests and surveys will be automated so all learners receive them at the same interval after receiving the learning materials. Non-responders will be emailed a series of reminders to complete the test and survey. Learners who do not complete the Diabetes Knowledge Test B and/or the Acceptability Survey will still be followed up and asked to complete the Practice Change Survey.

### Data collection and handling

Baseline data will be collected automatically when participants register online for the study. The following information will be recorded in the database: health professional role (doctor or nurse), preferred language to receive study materials (German or English), years since qualification, baseline Diabetes Knowledge Test score and whether they are registered users of the webservice univadis^® ^and/or BMJ Learning. Baseline information will be used to check comparability between study arms. Diabetes Knowledge Test B scores and follow-up questionnaires will be automatically collected online. Anonymised and blinded data will be exported in a format appropriate for statistical analysis.

### Interventions

Participants will not be told which group they have been allocated to. All participants will be given access to the same online Diabetes Learning Modules (see below) on a Learning Management System (LMS). An LMS is a technical platform which tracks all user behaviour including how long and how often materials are used, and also ensures that learners follow the materials in a fixed sequence. In addition, the intervention group will be administered a novel interactive learning tool, the DNAT. A link tracking system will capture which resources individual learners visit during the learning period. As the learners progress with the modules they will also be prompted to indicate which they completed and estimate the time they spent using them.

#### Diabetes Learning Modules

Participants in both groups have online access to the same Diabetes Learning Modules on the LMS. All the content of these learning modules is applicable to European practice and material comes from BMJ Learning, Excerpta Medica, a division of Elsevier Health Sciences, and the International Diabetes Federation and Elsevier Health Sciences. The modules include current evidence-based guidelines (particularly those of the European Association for the Study of Diabetes and the European Society of Cardiology) on diabetes, pre-diabetes and cardiovascular disease; important clinical areas and common difficulties in practice (Type 1, Type 2, diabetes in pregnancy and secondary causes of diabetes).

#### Diabetes Needs Assessment Tool (DNAT)

The DNAT is a new web-based diabetes interactive learning tool. It is a computerised adaptive test comprised of clinically rich case problems developed by a group of diabetologists/educationalists. The 253 DNAT test items have already been validated through use with a large number of learners with a range of ability registered on BMJ OnExamination. The items cover six categories: Principles of Diabetes (53 items), Lifestyle (49 items), Drug Treatment (64 items), CVS/Macrovascular (34 items), Acute Complications (21 items), Microvascular Complications (32 items). The DNAT can be completed over several sessions if required and takes the learner approximately 90 to 120 minutes to complete. Not all learners will see the same questions as the test adapts to knowledge level as individuals complete it. On completion of the DNAT, a personalised learning report is created for each learner identifying learning needs alongside individualised recommendations of the most appropriate Diabetes Learning Modules to meet those needs. At any stage this personalised report can be viewed listing the performance of the learner at that point.

### Outcome measures

#### Primary Outcome measure

##### Diabetes Knowledge Test

The Diabetes Knowledge Test contains 20 multiple choice questions (in the format of single best answer out of five possible options). There are two versions of the Diabetes Knowledge Test – Test A and Test B. All learners will receive the same baseline Diabetes Knowledge Test (Test A) prior to randomisation. Test B is a slightly different test of equal difficulty to be administered at the end of the learning period. The primary outcome will be Diabetes Knowledge Test scores at four months. Note-while the knowledge tests contain 20 items, only 19 items will be used in the knowledge test scores as one of the items in each test is a "rogue" item not on diabetes.

The Diabetes Knowledge Tests were developed from the same large pool of calibrated items as the DNAT but there is no overlap between the DNAT and the knowledge tests.

#### Secondary outcomes

##### Acceptability Survey

Immediately after the learning period, all learners will be administered a survey containing a series of questions about their level of satisfaction with the materials received and the acceptability of the learning formats. They will be asked about the perceived usefulness and usability of the materials. Participants in the intervention group will be asked additional specific questions about the acceptability of the DNAT.

##### Practice Change Survey

Five months after the start of the learning period, all learners will be administered a series of questions on self-reported changes in clinical practice as a result of the learning experience. They will be asked to give specific examples of any changes in their diabetes management. This will be used as an indicator of the transference of competence into clinical behaviour.

### Randomisation and allocation concealment

Eligible registered participants who complete Diabetes Knowledge Test A will be randomised to the control and intervention groups of the study. Optimal allocation with a ratio of 1:1 will be used. Randomisation will be balanced for language (English or German), ability (based on Diabetes Knowledge Test A scores), doctor vs nurse, and number of years since qualified at medical or nursing school, and whether they are registered users of the webservice univadis^® ^and/or BMJ Learning, using a minimisation technique.[18] The total sample of health professionals recruited will be divided into blocks of 24 and within each block a process of optimal allocation will be undertaken. This involves obtaining all possible allocations and calculating a balance statistic.[19] 1000 allocations with the greatest degree of balance will be identified and passed to a statistician within the South East Wales Trials Unit (SEWTU) at Cardiff University, (who is independent of the trial) who will randomly select a single allocation for each block. This will then be returned to the trial statistician (RP) and the study database manager informed of the allocations.

### Blinding

All outcome measures are tests and self-report surveys and will be administered automatically online. All data will be held in the online database. The statistical analysis of the trial data will be conducted by an independent statistician at SEWTU blinded to the group allocation of participants until the analyses are completed.

### Translation of study materials

All study materials were originally developed in English and subsequently translated into German. All recruitment and marketing material, communication to research participants, instructions on how to participate and website information were translated by an independent company and this was then reviewed by one of the bilingual members of the study team. The independent companies were selected by BMJ Group and contracted to do the work. While these companies do regular work for the pharmaceutical industry, they do not regularly work for MSD. The company did the work under their own editorial principles and MSD had no influence on the work that they produced. The DNAT and Diabetes Knowledge Tests underwent a more rigorous multi-step process. Content was translated by an independent company (from English to German) and the translated text from this company was then sent to a second independent company for proofing. The text was then medically reviewed by bilingual members of the research team and the texts finalised.

### Sample size

We estimated that for a moderate effect size of 0.4 we needed a minimum of 100 participants in each arm (at a significance level of 0.5 and power of 80%). For an effect size of less than that, e.g. 0.3 the sample size increases to 176 per group. These needed to be inflated to account for drop out.

Since recruitment was to take place via online and print advertisement and registration, recruitment rates, learning material uptake and drop out were unknown, we decided to allow up to 1000 participants per country to complete the registration and baseline test until the cut off date for study recruitment. Obtaining a larger sample size than stated above will provide greater power for smaller effect sizes and allow the country subgroup analyses to have more power.

### Statistical Analysis

All data will be checked during data cleaning for outlier data. Range checks from the online data collection will eliminate any out of range data. Any observed outliers will be checked with the database management team. Intention to treat analysis will be carried out for the primary analysis. Participants will be encouraged to complete Diabetes Knowledge Test B even if they have not spent as much time learning as they had planned. Complete case analysis will be used as a secondary analysis.

Summary statistics on participant assessment, eligibility, recruitment, withdrawal and dropout will be collated for both arms of the trial and form the CONSORT flow diagram for clinical trial reporting. The primary analysis will use Analysis of Covariance (ANCOVA) to compare the follow-up diabetes knowledge test scores between trial arms adjusting for baseline score as a covariate. This analysis makes distributional assumptions about the data and these will be checked prior to analysis. Variables used in the balancing algorithm will also be considered for inclusion as covariates. Secondary outcome analysis will compare the acceptability and practice change outcomes between trial arms. T-tests and Chi square tests will be used where appropriate for questionnaire items.

#### Missing data

In the first instance missing follow-up Diabetes Knowledge Test Score data (Test B) will be assumed to be missing at random. Baseline checks will be carried out to test this assumption. Missing follow-up Diabetes Knowledge Test Scores (Test B) will be assumed to have remained unchanged for the intention to treat analysis. A complete case analysis will also be carried out excluding those missing follow-up test scores. A sensitivity analysis will be carried out to assess the difference between these analyses.

#### Subgroup analysis

Planned subgroup analyses involve the investigation of the learning outcomes within language group. This will be achieved by the addition of an interaction term to the primary analysis.

### Trial organisation

The principal investigator will co-ordinate the trial with the assistance of the Chief Information Officer (CIO) at BMJ OnExamination and chair regular steering group meetings with the research co-investigators. The co-investigators will regularly monitor the study's progress. SEWTU will provide input at management group level for the design and conduct of the study together with statistical input in the design of the websites to ensure quality data collection. SEWTU will validate the data collection and randomisation together with the CIO from OnExamination. The CIO is responsible for the management, recording, and storage of data. The study protocol is managed centrally by the CIO and any protocol deviations will be documented and reviewed by the steering group at regular intervals.

#### Data Quality Assurance

All study tests and surveys will be administered to participants online and the data collected centrally on an independent platform. Participants will be asked for their email address at enrolment and will be prevented from enrolling more than once using the same email address. This should prevent the majority of duplication. Shortly after randomisation a check will be made for those that enrolled with identical firstnames, surnames, and language choice but different email addresses. The first registered email address that had a completed baseline Diabetes Knowledge Test will be assigned as their account and any other email addresses disabled. We will then write to these participants to tell them that we noticed they had registered more than once and that they should use the selected email address from now on as we will disable the other(s).

#### Publication Policy

We will submit the results of the trial for publication in a biomedical journal irrespective of outcome.

#### Timetable

February to March 2009: Recruitment of learners. Registered learners gave consent and were administered Diabetes Knowledge Test A

25^th ^to 31^st ^March 2009: Stratified randomisation

1^st ^April – 31^st ^July 2009: 4-month learning period

1^st ^August 2009: Administration of Diabetes Knowledge Test B and the Acceptability Survey

1^st ^September 2009: Administration of the Practice Change Survey

25^th ^September 2009: Close of data collection

26^th ^September 2009: Data to be given to independent statisticians for analysis

## Abbreviations

DNAT: Diabetes Needs Assessment Tool; BMJ Group: BMJ Publishing Group; SEWTU: South East Wales Trials Unit; Cardiff University

## Competing interests

SS is a full time employee of the BMJ Group. She regularly conducts research into all aspects of publishing for BMJ Group and will not benefit financially from the outcome of this study.

DJ is a Director of BMJ OnExamination. He is the Principal Investigator for the study and developed the DNAT and Diabetes Knowledge Tests from test items generated by BMJ OnExamination. He will not benefit financially from the success of the DNAT if it is shown to be effective.

RP: No competing interests.

KW is editor of BMJ Learning and works for the BMJ Group. He is paid a fixed salary.

CP is Chief Information Officer for BMJ OnExamination. He developed the DNAT and Diabetes Knowledge Tests from test items generated by BMJ OnExamination. He also developed the websites for the study. He will not benefit financially from the success of the DNAT if it is shown to be effective.

TK is a full time employee of MSD RBSC GmbH. He helped design the study and helped with the marketing campaign to promote it to potential participants. He will not have access to the data until after it has been analysed by researchers at Cardiff University and then will only be given access to anonymised data.

GA is a full time employee of Merck Sharp & Dohme Limited. He helped design the study and helped with the set up of the Learning Management System and the provision of the online learning modules. He will not have access to the data until after it has been analysed by researchers at Cardiff University and then will only be given access to anonymised data

DO has conducted lectures, nationally and internationally and served as a consultant for a number of pharmaceutical companies for which he has received honoraria. He will not benefit financially from the success of the DNAT if it is shown to be effective.

## Authors' contributions

SS formulated the research question, designed the study, helped with trial management and wrote the first draft of this manuscript. DJ is Principal Investigator and guarantor of this paper. DJ helped formulate the research question, developed the DNAT and helped revise the manuscript. CP developed the DNAT and the technology to run the study and deliver the learning interventions, and contributed to the study design. RP contributed to the study design, formulated the statistical analysis plan and helped draft this manuscript. KW contributed to the study design and helped revise the manuscript. TK contributed to the study design, managed the recruitment plan for the German participants, and helped revise the manuscript. GA contributed to the study design and helped in the technical provision of the learning interventions and the Learning Management System. DO contributed to the study design and helped revise the manuscript. All authors read and approved the final manuscript.

## Funding

Merck Sharp and Dohme Regional Business Support Center (MSD RBSC) GmbH (Germany) gave BMJ Group £110,000 to help fund this study. All other expenses for the study, except translation costs, were paid by BMJ Group. While staff at MSD RBSC GmbH were involved with designing the study and the recruitment campaigns for learners, they will not have access to the trial data until the end of the study and will not be involved in the statistical analysis or interpretation of data. All statistical analysis will be performed by independent researchers at Cardiff University and they will be blinded to group allocation.
